# Ultra-Early OCT Changes After Intravitreal Injection: Evidence Consistent with Transient Mechanical Compression

**DOI:** 10.3390/vision10020035

**Published:** 2026-06-14

**Authors:** Yehya Tlaiss, John Warrak, Elias Warrak

**Affiliations:** 1 Department of Ophthalmology, University of Balamand, Beirut 1107 2020, Lebanon; john.warrak@std.balamand.edu.lb (J.W.);; 2 Advanced Eye Care Center, University of Balamand, Beirut 1107 2020, Lebanon

**Keywords:** intravitreal injection, optical coherence tomography, macular edema, intraocular pressure, mechanical compression, anti-VEGF

## Abstract

(1) Background: Ultra-early optical coherence tomography (OCT) changes following intravitreal injection may reflect transient mechanical compression rather than pharmacologic effects; however, this temporal profile has not been rigorously characterised with appropriate statistical methodology. (2) Methods: In this prospective observational study, 40 eyes of 40 consecutive patients (one per patient) with macular edema secondary to neovascular age-related macular degeneration (nAMD), diabetic macular edema (DME), or chronic central serous retinopathy (CSR) underwent intravitreal bevacizumab (*n* = 35) or triamcinolone acetonide (*n* = 5). Goldmann applanation tonometry and spectral-domain OCT were performed at baseline, 2–5 min, 15 ± 5 min, 24 h, and 48 h post-injection. Repeated-measures ANOVA with Greenhouse–Geisser correction, linear regression, and Spearman rank correlation were applied. (3) Results: Central subfield thickness (CST) decreased markedly at 15 ± 5 min (mean −24.8 ± 11.5%; 95% CI: −28.5% to −21.1%; *p* < 0.001; partial η^2^ = 0.70), with near-complete rebound by 48 h (−1.0%; *p* = 0.400). Peak intraocular pressure (IOP) elevation correlated with CST reduction (Spearman r_s_ = 0.61; 95% CI: 0.39–0.77; *p* < 0.001), and baseline CST predicted thinning magnitude (R^2^ = 0.52; *p* < 0.001). (4) Conclusions: Ultra-early OCT thinning after intravitreal injection is consistent with transient mechanical compression. Retinal thickness measurements within 48 h post-injection should be interpreted with caution when assessing treatment response, as early anatomic reduction may not reflect pharmacologic efficacy.

## 1. Introduction

Intravitreal pharmacotherapy is the cornerstone of management for retinal diseases associated with macular edema, including neovascular age-related macular degeneration (nAMD) and diabetic macular edema (DME). Repeated intravitreal injections of anti-vascular endothelial growth factor (anti-VEGF) agents such as bevacizumab constitute first-line therapy in these conditions and are associated with significant visual and anatomic improvement [1]. Intravitreal corticosteroids, including triamcinolone acetonide, are also used in selected cases of DME or refractory disease, although their role in nAMD is limited by lower efficacy and a higher risk of adverse effects [1]. The therapeutic goal of these interventions is a reduction in retinal vascular permeability and inflammatory activity, ultimately leading to the resolution of intraretinal and subretinal fluid.

Optical coherence tomography (OCT) has become the standard noninvasive modality for monitoring treatment response, allowing precise quantification of macular thickness and fluid dynamics. In routine clinical practice, the anatomic response to intravitreal therapy is typically assessed weeks after injection, when pharmacologic effects are expected to predominate. However, several studies have reported OCT-detectable changes occurring within hours after intravitreal injection [2]. The mechanism underlying these ultra-early changes and their clinical significance remain incompletely understood.

One proposed explanation is that intravitreal injection produces an acute mechanical effect related to sudden increases in intraocular volume and pressure. Injection of 0.05–0.1 mL of fluid into the vitreous cavity commonly results in a transient elevation in IOP [3], which may mechanically compress the retina and redistribute intraretinal and subretinal fluid. This volumetric compression could produce an apparent reduction in macular thickness that is independent of the pharmacologic action of the injected agent. In contrast, the biological effects of anti-VEGF agents and corticosteroids—mediated through alterations in vascular permeability, endothelial junctions, and inflammatory signalling—are not expected to manifest within the first hours to days after injection [4,5].

Distinguishing between mechanical and pharmacologic components is clinically important. Marked OCT improvement immediately after injection may falsely suggest rapid therapeutic efficacy, while the absence of early thinning may be misinterpreted as treatment failure. Prior reports have demonstrated significant macular thickness reductions shortly after intravitreal triamcinolone, with less consistent early changes following anti-VEGF therapy [2,6], supporting the hypothesis that injection volume and pressure dynamics substantially influence early OCT measurements. Importantly, Gunay et al. demonstrated that first-day anti-VEGF results do not reliably predict long-term anatomic outcomes in macular edema secondary to vascular diseases [7], further questioning the biological relevance of ultra-early measurements.

**Figure 1:**
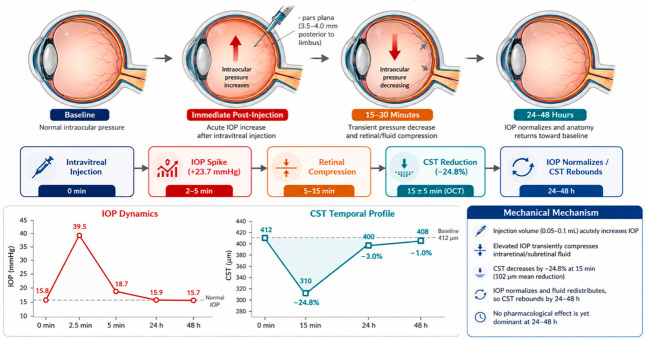
Proposed mechanism of ultra-early OCT thinning following intravitreal injection. Acute IOP elevation compresses intraretinal fluid, producing apparent macular thinning that reverses as IOP normalises. Lower insets show IOP and CST temporal profiles. IOP, intraocular pressure; CST, central subfield thickness; OCT, optical coherence tomography.

Despite these observations, the ultra-early temporal profile of macular thickness changes following intravitreal injection has not been systematically characterised using standardised OCT timing and concurrent IOP assessment, nor analysed with appropriate repeated-measures methodology. The present prospective observational study was therefore designed to characterise ultra-early OCT changes following intravitreal injection and to examine whether these changes are consistent with transient mechanical compression rather than early pharmacologic therapeutic effects. Prior studies have documented that IOP rises sharply within 2–5 min of injection, then declines substantially within 10–15 min and returns toward baseline within 30–60 min [3]; accordingly, the 15 ± 5 min post-injection window was selected as the primary OCT acquisition time point to capture a clinically feasible ultra-early measurement while IOP remains above baseline. The 48 h time point was chosen as a reference for near-complete resolution, given that pharmacologic agents are not expected to produce measurable anatomic effects within this window [4,5]. The proposed sequence of transient mechanical compression and subsequent rebound is schematically illustrated in Figure 1.

## 2. Materials and Methods

### 2.1. Study Design and Participants

This prospective observational study was conducted at a tertiary eye care centre (Advanced Eye Care Center, Saint George Hospital University Medical Center, Beirut, Lebanon). Of 52 consecutive patients screened during the enrolment period, 40 were enrolled and constituted the final study population. Twelve patients were excluded: four had significant media opacity precluding adequate OCT quality, five had received an intravitreal injection within the preceding six weeks, two had vitreomacular traction on baseline OCT, and one declined participation. No patients were lost to follow-up and no measurements were missing at any time point.

Strictly one eye per patient was enrolled to avoid inter-eye correlation and preserve the statistical independence of observations. Where both eyes were potentially eligible, the eye with higher clinical priority (as determined by the treating physician) was selected.

Eligible diagnoses included nAMD, DME, and chronic CSR. Patients were either treatment-naïve or had completed a minimum washout of six weeks from any prior intravitreal therapy. Eyes with significant media opacity, vitreomacular traction, epiretinal membrane, or other retinal comorbidities likely to affect macular thickness measurements were excluded. All participants provided written informed consent for intravitreal treatment and the additional study measurements.

### 2.2. Sample Size Determination

Sample size was determined by a priori power analysis using G*Power (version 3.1.9.7, Heinrich Heine Universität Düsseldorf). Based on prior studies reporting within-subject standard deviations of approximately 90–100 μm for CST in macular edema cohorts [2,6], and assuming a minimum clinically relevant mean CST change of 30 μm at the ultra-early time point with a repeated-measurement correlation of 0.70, the G*Power input parameters were: effect size f = 0.40 (corresponding to partial η^2^ ≈ 0.14; a medium-to-large within-subject effect), within-subject SD = 95 μm, and correlation among repeated measures = 0.70. Under these assumptions, at least 34 eyes were required to achieve 80% power at a two-tailed significance level of α = 0.05 using repeated-measures ANOVA. An enrolment target of 40 eyes was set to account for potential scan failures and to provide additional power for secondary regression analyses.

### 2.3. Ethical Approval and Consent

The study was approved by the Institutional Review Board of the University of Balamand on 1 February 2025 (Approval Reference: IRB-REC/o/025-08/0225). Written informed consent was obtained from all participants prior to enrolment. The study was conducted in accordance with the tenets of the Declaration of Helsinki.

### 2.4. Intravitreal Injection Procedure

All injections were performed by the same surgeon (E.W.) using standard aseptic technique. Thirty-five eyes received intravitreal bevacizumab (1.25 mg/0.05 mL) and five received triamcinolone acetonide (4 mg/0.1 mL), via a 30-gauge needle through the pars plana (3.5 mm posterior to the limbus in pseudophakic eyes; 4.0 mm in phakic eyes). Immediately following needle withdrawal, a sterile cotton-tip applicator was applied to the sclera at the injection site with gentle digital pressure for approximately 30 s to minimise medication reflux. This manoeuvre was standardised across all participants. It should be noted that post-injection scleral pressure with a cotton-tip applicator may itself transiently influence IOP dynamics and retinal compression, although this is a routine component of standard intravitreal injection technique and was applied uniformly, it represents a potential confounder that cannot be fully disentangled from the effect of injection volume alone. Ocular perfusion was assessed clinically by hand-motion testing at the conclusion of each procedure. No study eye required anterior chamber paracentesis.

### 2.5. Intraocular Pressure Measurement

IOP was measured using Goldmann applanation tonometry (Haag-Streit AT 900, Haag-Streit AG, Köniz, Switzerland) by the same certified examiner (Y.T.) at five predefined time points: baseline (pre-injection), 2–5 min post-injection, 15 ± 5 min post-injection, 24 h, and 48 h. The standardised measurement sequence was as follows: (a) the cotton-tip applicator pressure was applied immediately after needle withdrawal and released after approximately 30 s; (b) the 2–5 min IOP measurement was performed after cotton-tip pressure was released, with the first tonometric reading taken at approximately 2 min post-needle withdrawal; and (c) the tonometrist was not masked to injection status but was not informed of concurrent OCT results at the time of measurement. Topical proparacaine 0.5% was used for anaesthesia prior to each tonometric reading; corneal surface irregularities or patient discomfort were noted as potential sources of variability in the early post-injection readings.

### 2.6. OCT Imaging Protocol

Spectral-domain OCT (SD-OCT) imaging was performed using the Heidelberg Spectralis (Heidelberg Engineering, Heidelberg, Germany) at four standardised time points: baseline, 15 ± 5 min post-injection, 24 h (±2 h), and 48 h post-injection. All scans were acquired in dense-raster macular mode with automatic real-time averaging (≥16 frames) and eye-tracking. Central subfield thickness (CST), defined as the mean retinal thickness within the central 1 mm ETDRS subfield, was the primary OCT outcome. Total macular volume within the central 6 mm area was a secondary anatomic measure. Automated retinal layer segmentation was performed by the Spectralis built-in segmentation software. All scans were reviewed for segmentation accuracy by a single trained grader (Y.T.) who was masked to the time point and clinical outcome. Scans with segmentation errors (layer boundary misplacement exceeding 10 μm) were manually corrected by the same grader; scans that could not be adequately corrected due to motion artefact or persistent boundary failure were to be excluded. Motion artefacts were identified based on the presence of horizontal banding or vessel doubling on the en face image, and any affected scan was re-acquired immediately. 

### 2.7. Statistical Analysis

Continuous variables are reported as mean ± standard deviation (SD). Normality was confirmed using the Shapiro–Wilk test (W > 0.95, *p* > 0.05 for all variables). The primary analysis of CST change across four time points was performed using repeated-measures ANOVA with Greenhouse–Geisser correction for sphericity (Mauchly’s W = 0.62, *p* < 0.001; ε = 0.70). IOP dynamics across five time points were analysed analogously. Pairwise post hoc comparisons with Bonferroni correction were performed. Effect sizes are reported as partial eta-squared (η^2^). Linear regression quantified the association between baseline CST and ultra-early CST percentage reduction (unstandardised β, standardised β, 95% CI, R^2^ reported). The association between peak IOP elevation and ultra-early CST reduction was assessed using Spearman rank correlation (r_s_; 95% CI via bootstrap with 1000 iterations). Subgroup analyses by treatment agent and diagnosis were exploratory only. All tests were two-tailed (α = 0.05). Analyses were performed using IBM SPSS Statistics v25.0 (IBM Corp., Armonk, NY, USA).

Artificial intelligence (AI) assistance was used for manuscript language editing and figure generation. All AI-generated content was reviewed and edited by the authors, who take full responsibility for the final content.

## 3. Results

### 3.1. Patient and Ocular Characteristics

Forty eyes from 40 patients were included (Table 1). Mean age was 64.2 ± 9.8 years (range, 45–81), with 22 males (55%). The cohort comprised 20 eyes with nAMD (50%), 15 with DME (37.5%), and 5 with CSR (12.5%). Thirty-five eyes received bevacizumab and five received triamcinolone acetonide. All 40 eyes completed all scheduled OCT and IOP measurements; no data were missing and no patients were lost to follow-up.

Mean baseline CST was 412 ± 95 μm (range, 300–650 μm). Eyes with DME had the highest mean baseline CST (445 ± 108 μm), followed by nAMD (398 ± 82 μm) and CSR (352 ± 65 μm). Mean baseline IOP was 15.8 ± 2.6 mmHg.

### 3.2. Intraocular Pressure Dynamics

**Figure 2:**
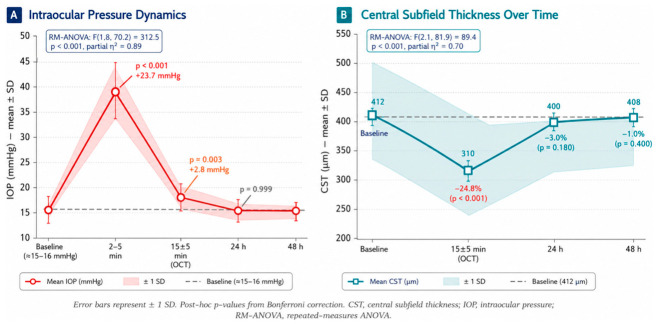
Temporal profiles of IOP (Panel (**A**)) and CST (Panel (**B**)) following intravitreal injection. Panel (**A**): mean IOP (±1 SD) at five time points; dashed line indicates baseline (15.8 mmHg). Panel (**B**): mean CST (±1 SD) at four OCT time points; dashed line indicates baseline (412 μm). Percentage changes from baseline and Bonferroni-corrected *p*-values are annotated at each post-injection time point. Error bars represent ±1 SD. All pairwise comparisons are Bonferroni-corrected. RM-ANOVA: IOP, F(1.8, 70.2) = 312.5, ε = 0.70, partial η^2^ = 0.89; CST, F(2.1, 81.9) = 89.4, ε = 0.70, partial η^2^ = 0.70 (both *p* < 0.001). Diagnosis- and agent-level subgroup patterns shown are exploratory and underpowered; they should not be interpreted as confirmatory. IOP, intraocular pressure; CST, central subfield thickness; SD, standard deviation; RM-ANOVA, repeated-measures ANOVA; ε, Greenhouse–Geisser epsilon.

Repeated-measures ANOVA revealed a highly significant main effect of time on IOP [F(1.8, 70.2) = 312.5, *p* < 0.001, partial η^2^ = 0.89]. Post hoc Bonferroni comparisons confirmed a peak elevation at 2–5 min post-injection (mean +23.7 mmHg; 95% CI: +21.0 to +26.4; *p* < 0.001). A modest but statistically significant residual elevation persisted at 15 ± 5 min (+2.8 mmHg; 95% CI: +1.7 to +3.9; *p* = 0.003), coinciding with the OCT acquisition time point. IOP at 24 and 48 h did not differ significantly from baseline (both *p* = 0.999). No eye required anterior chamber paracentesis or pharmacologic IOP-lowering intervention. The temporal profile of IOP dynamics is illustrated in Table 2 and Figure 2A.

### 3.3. Ultra-Early OCT Changes

Repeated-measures ANOVA demonstrated a highly significant main effect of time on CST [F(2.1, 81.9) = 89.4, *p* < 0.001, partial η^2^ = 0.70]. Post hoc Bonferroni comparisons confirmed a large and highly significant reduction at 15 ± 5 min (mean reduction: 102 μm; 95% CI: 84–120 μm; percentage reduction: 24.8 ± 11.5%; 95% CI: −28.5% to −21.1%; *p* < 0.001). Individual eye reductions ranged from approximately 10% to 40%. A corresponding reduction in total macular volume was observed, consistent with a global transient decrease rather than localised segmentation artefact. Regarding segmentation quality: no scans were excluded due to motion artefact or irrecoverable segmentation failure. Fourteen scans (8.8% of all post-injection acquisitions) required minor manual segmentation correction; the magnitude of CST change was unchanged when these corrected scans were included versus when sensitivity analyses were performed using only scans requiring no manual correction (difference < 1.5 μm; *p* > 0.80).

**Figure 3:**
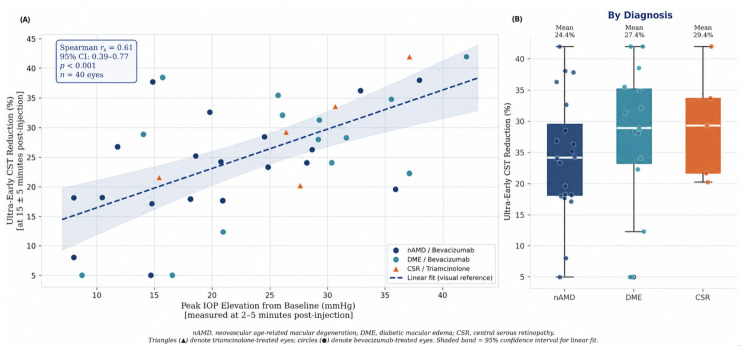
(**A**) Association between peak IOP elevation and ultra-early CST reduction. Each point represents one eye, colour-coded by diagnosis (navy: nAMD; teal: DME; orange: CSR) and marker-coded by agent (●: bevacizumab; ▲: triamcinolone). Dashed line: least-squares regression fit; shaded band: 95% CI. Spearman r_s_ = 0.61 (95% CI: 0.39–0.77; *p* < 0.001; *n* = 40). (**B**) Distribution of ultra-early CST reduction stratified by diagnosis; boxes represent IQR, whiskers extend to 1.5× IQR, with individual data points overlaid. IOP, intraocular pressure; CST, central subfield thickness; nAMD, neovascular age-related macular degeneration; DME, diabetic macular edema; CSR, central serous retinopathy; IQR, interquartile range.

The magnitude of ultra-early CST reduction was significantly predicted by baseline CST: for each 10 μm increase in baseline macular thickness, ultra-early thinning increased by approximately 1.9 percentage points (unstandardised β = 0.19%/μm; 95% CI: 0.14–0.24; standardised β = 0.72; R^2^ = 0.52; F(1, 38) = 41.2; *p* < 0.001). Peak IOP elevation correlated moderately and significantly with ultra-early CST reduction (Spearman r_s_ = 0.61; 95% CI: 0.39–0.77; *p* < 0.001). These associations are summarised in Table 3 and illustrated in Figure 3.

### 3.4. Temporal Evolution of Macular Thickness

At 24 h post-injection, substantial rebound was observed: mean CST reduction was only −3.0 ± 7.5% (95% CI: −5.4% to −0.6%), which did not reach statistical significance after Bonferroni correction (*p* = 0.180). By 48 h, CST had returned to near-baseline (−1.0 ± 6.8%; 95% CI: −3.2% to +1.2%; *p* = 0.400). This rebound pattern was consistent across all diagnostic subgroups. The temporal evolution of CST is presented in Table 4 and Figure 2B.

### 3.5. Regression and Correlation Analyses

Baseline CST accounted for 52% of the variance in ultra-early thinning magnitude (R^2^ = 0.52; Table 3). The moderate Spearman correlation between peak IOP elevation and CST reduction (r_s_ = 0.61) supports a pressure-dependent mechanism, though unexplained variance suggests additional contributors such as vitreous compliance and pre-existing fluid compartmentalisation.

### 3.6. Treatment Subgroup Analysis

Eyes treated with bevacizumab and triamcinolone acetonide demonstrated broadly similar temporal patterns of ultra-early CST reduction and subsequent rebound. Steroid-treated eyes tended to exhibit numerically larger early reductions, potentially reflecting the larger injection volume (0.1 mL vs. 0.05 mL), though no sustained CST differences were observed at 48 h. Given that only five eyes received triamcinolone acetonide, this subgroup is insufficiently powered for formal statistical comparison; these findings are strictly exploratory. Diagnostic imbalance between treatment groups precludes definitive group-level inference.

### 3.7. Safety Outcomes

No serious ocular adverse events occurred. Mild transient ocular discomfort and blurred vision were commonly reported on the day of injection and resolved spontaneously. No cases of endophthalmitis, retinal tear, or sustained IOP elevation were observed.

## 4. Discussion

In this prospective observational study of 40 eyes, we demonstrate that ultra-early macular thickness reductions on OCT following intravitreal injection are transient and consistent with a predominantly mechanical origin rather than early pharmacologic therapeutic effect. A marked decrease in CST was observed at 15 ± 5 min post-injection, with rapid rebound and near-complete return to baseline by 48 h. Repeated-measures ANOVA confirmed the statistical robustness of this temporal pattern (partial η^2^ = 0.70), and the moderate correlation between peak IOP elevation and thinning magnitude (Spearman r_s_ = 0.61) provides mechanistic support—though not definitive proof—for a pressure-dependent compressive mechanism.

The timing of these changes is biologically informative. Anti-VEGF agents exert their therapeutic effects through modulation of vascular permeability and endothelial signalling, processes that typically evolve over days to weeks rather than minutes to hours [2,5]. Pharmacokinetic modelling demonstrates that even after peak intraretinal concentration is reached within 24–48 h of injection, the downstream effects on junctional proteins, fluid transport, and oedema resolution manifest gradually over subsequent days [5]. Similarly, corticosteroids may exhibit some non-genomic anti-inflammatory actions, but sustained anatomic effects are generally not expected within the first 24–48 h [4]. The absence of durable OCT improvement at 48 h across both treatment groups therefore argues against a meaningful pharmacologic contribution during this ultra-early window.

The most plausible mechanism is acute volume- and pressure-related compression of intraretinal and subretinal fluid. Kotliar et al. demonstrated substantial immediate IOP elevations following intravitreal injection, proportional to injected volume [3]. In the present study, IOP was markedly elevated at 2–5 min (+23.7 mmHg) and remained modestly but significantly elevated at 15 ± 5 min (+2.8 mmHg; *p* = 0.003), the time point at which OCT was acquired. This temporal overlap between residual IOP elevation and ultra-early OCT thinning, combined with the positive IOP–CST correlation, is consistent with—though does not definitively prove—a pressure-mediated compressive mechanism. Eyes with greater baseline macular thickening demonstrated proportionally larger early reductions (R^2^ = 0.52), suggesting that more fluid-laden maculae are more susceptible to mechanical compression. An important temporal limitation must be acknowledged: OCT was acquired at 15 ± 5 min post-injection, when IOP had already partially normalised (+2.8 mmHg), rather than at the true IOP peak at 2–5 min (+23.7 mmHg). This temporal offset means that the observed CST reduction likely underestimates the maximal compressive effect; had OCT been acquired at peak IOP, larger thinning magnitudes would be expected. Future studies incorporating real-time or continuous OCT acquisition immediately post-injection would be needed to characterise the full extent of this effect [8]. Additionally, the standardised application of scleral cotton-tip pressure after needle withdrawal, while routine, may independently influence IOP dynamics and retinal compression to an unknown degree and should be acknowledged as a potential confounder in the mechanistic interpretation.

It is important to note that the study design—lacking a sham or volume-only injection control arm, which was not feasible on ethical grounds—limits mechanistic inference. The interpretation that early changes “support” a mechanical mechanism is therefore appropriate, and definitive causal language such as “proves” or “dominant mechanism” has been deliberately avoided. Furthermore, the absence of lasting differences at 48 h does not prove the absence of early biological effects; it indicates only that the observed primary pattern is consistent with a mechanical effect. As Gunay et al. have noted, first-day anti-VEGF results do not predict long-term outcomes [7], underscoring that ultra-early measurements reflect non-pharmacological processes.

Our findings are broadly consistent with the prior literature. He et al. reported significant reductions in central macular thickness within hours of intravitreal triamcinolone and bevacizumab injection [2]. Sonoda et al. demonstrated early thinning following intravitreal triamcinolone, with larger effects after higher-volume injections [6]. By demonstrating near-complete rebound by 48 h with rigorous repeated-measures analysis, the present study extends this evidence base. The heterogeneity of the study population—three diagnoses and two treatment modalities—warrants caution in interpreting subgroup patterns. The highly unbalanced design (35 bevacizumab vs. 5 triamcinolone) precludes definitive treatment-group comparisons.

The clinical implications are significant. Ultra-early OCT imaging within 24–48 h after intravitreal injection may be misleading if used to assess therapeutic response. These findings reinforce established clinical practice in which treatment efficacy is evaluated at four-to-six-week follow-up intervals [1]. Beyond routine care, the results are particularly relevant for early-phase clinical trials evaluating novel intravitreal agents using early OCT endpoints: without accounting for injection-related mechanical effects, apparent anatomic improvements may be incorrectly attributed to drug efficacy [9]. From a translational perspective, the characterisation of these mechanical IOP-mediated effects on retinal structure is consistent with the emerging evidence that ocular biomechanical forces influence cellular and stromal responses relevant to drug delivery and tissue remodelling [8]. As a practical recommendation for clinicians: OCT should not be scheduled within 48 h of intravitreal injection for the purpose of treatment-response assessment; if early imaging is clinically required, the time elapsed since injection and the IOP at the time of imaging should be documented and considered when interpreting anatomic findings. Additionally, the findings may not be fully generalisable to other anti-VEGF agents (e.g., ranibizumab, aflibercept, faricimab) or to injection techniques using different needle gauges or volumes, which should be addressed in future studies.

### Limitations

Several limitations should be acknowledged. First, the sample size was modest and the triamcinolone subgroup (*n* = 5) was insufficient for reliable comparative inference; all conclusions regarding this arm are strictly exploratory and should not be generalised to corticosteroid injections more broadly. Second, the cohort included heterogeneous disease entities and treatment agents, limiting disease-specific mechanistic inference; subgroup analyses by diagnosis and agent are exploratory and underpowered. Third, no sham or volume-only control arm was included due to ethical considerations, precluding the direct isolation of purely mechanical effects; the observed OCT changes may therefore reflect multiple injection-related factors, including injected volume, needle entry trauma, scleral indentation, IOP spike, and potentially drug-specific biology. Fourth, OCT imaging was not performed within the immediate 2–5 min post-injection window when IOP is maximally elevated, preventing characterisation of the true peak OCT change and likely resulting in underestimation of the maximal compressive effect; future studies should incorporate real-time OCT acquisition at the point of peak IOP. Fifth, the application of a cotton-tip applicator after needle withdrawal may have independently influenced IOP dynamics and retinal compression to an unknown degree, representing a potential confounder that cannot be fully separated from injection volume effects. Sixth, a linear mixed-effects model may be more appropriate than repeated-measures ANOVA to accommodate the possibility of missing data, unequal intervals, and subject-level random effects; this should be considered in future work. Seventh, the present findings may not be fully generalisable to other anti-VEGF agents (e.g., ranibizumab, aflibercept, faricimab), different injection volumes, or alternative injection techniques. Eighth, functional outcome measures were not assessed due to the short follow-up interval.

## 5. Conclusions

Ultra-early OCT thinning observed after intravitreal injection is consistent with transient mechanical compression rather than pharmacologic therapeutic effect, as evidenced by the temporal concordance of IOP dynamics and CST changes, the significant correlation between peak IOP elevation and thinning magnitude (r_s_ = 0.61), and near-complete rebound by 48 h. Retinal thickness measurements obtained within the first 48 h after injection should be interpreted with caution, as early anatomic reduction does not reliably reflect durable disease modification. Recognition of this phenomenon may improve clinical decision-making, prevent misinterpretation of early OCT findings, and inform the design of future clinical trials evaluating intravitreal therapies, particularly those using ultra-early anatomic endpoints.

## Figures and Tables

**Table 1:** Baseline demographic and ocular characteristics.

Characteristic	Value
Number of eyes (patients)	40 (40)
Age, years (mean ± SD, range)	64.2 ± 9.8 (45–81)
Sex, *n* (%)	Male: 22 (55%); Female: 18 (45%)
Diagnosis, *n* (%)	nAMD: 20 (50%); DME: 15 (37.5%); CSR: 5 (12.5%)
Treatment received, *n* (%)	Bevacizumab 1.25 mg/0.05 mL: 35 (87.5%) Triamcinolone acetonide 4 mg/0.1 mL: 5 (12.5%)
Baseline CST, μm (mean ± SD, range)	412 ± 95 (300–650) nAMD: 398 ± 82; DME: 445 ± 108; CSR: 352 ± 65
Baseline IOP, mmHg (mean ± SD, range)	15.8 ± 2.6 (11–21)
BCVA range at baseline	20/40–20/200
One eye included per patient	Yes

nAMD, neovascular age-related macular degeneration; DME, diabetic macular edema; CSR, central serous retinopathy; CST, central subfield thickness; IOP, intraocular pressure; BCVA, best-corrected visual acuity; SD, standard deviation.

**Table 2:** Intraocular pressure dynamics following intravitreal injection.

Time Point	IOP, mmHg Mean ± SD (Range)	Change from Baseline, mmHg (95% CI)	*p*-Value *
Baseline (pre-injection)	15.8 ± 2.6 (11–21)	Reference	—
2–5 min post-injection	39.5 ± 8.4 (26–58)	+23.7 (+21.0 to +26.4)	<0.001
15 ± 5 min post-injection	18.6 ± 3.1 (13–25)	+2.8 (+1.7 to +3.9)	0.003
24 h post-injection	15.9 ± 2.7 (11–22)	+0.1 (−0.8 to +1.0)	0.999
48 h post-injection	15.7 ± 2.5 (12–21)	−0.1 (−0.9 to +0.7)	0.999

* Repeated-measures ANOVA with Greenhouse–Geisser correction: F(1.8, 70.2) = 312.5, *p* < 0.001, ε = 0.70, partial η^2^ = 0.89. Post hoc *p*-values from Bonferroni correction. IOP, intraocular pressure; CI, confidence interval; ε, Greenhouse–Geisser epsilon. For the 2–5 min IOP measurement, the range (26–58 mmHg) suggests a skewed distribution; median and interquartile range were 38.5 mmHg (IQR: 33.0–45.0 mmHg) for reference alongside the reported mean ± SD.

**Table 3:** Summary of regression and correlation analyses for ultra-early CST reduction.

Association	Coefficient	95% CI	Model Fit	*p*-Value
Baseline CST (μm) → Ultra-early CST % reduction [Linear regression]	β = 0.19%/μm (β_std = 0.72)	0.14–0.24	R^2^ = 0.52 F(1, 38) = 41.2	<0.001
Peak IOP elevation (mmHg) → Ultra-early CST % reduction [Spearman correlation]	r_s_ = 0.61	0.39–0.77	—	<0.001

The regression slope (β = 0.19%/μm) indicates that, for every 10 μm increase in baseline macular thickness, the magnitude of ultra-early thinning increases by approximately 1.9 percentage points; this means that patients with thicker, more oedematous retinas at baseline will exhibit proportionally greater mechanical thinning after injection. β, unstandardised regression coefficient; β_std, standardised regression coefficient; r_s_, Spearman rank correlation coefficient; 95% CI for r_s_ computed by bootstrap (1000 iterations). CST, central subfield thickness; IOP, intraocular pressure; CI, confidence interval.

**Table 4:** Temporal changes in central subfield thickness following intravitreal injection.

Time Point	CST, μm Mean ± SD (Range)	% Change from Baseline Mean ± SD (95% CI)	*p*-Value *
Baseline	412 ± 95 (300–650)	Reference	—
15 ± 5 min post-injection	310 ± 85 (220–520)	−24.8 ± 11.5 (−28.5 to −21.1)	<0.001
24 h post-injection	400 ± 92 (290–640)	−3.0 ± 7.5 (−5.4 to −0.6)	0.180
48 h post-injection	408 ± 94 (295–645)	−1.0 ± 6.8 (−3.2 to +1.2)	0.400

* Repeated-measures ANOVA with Greenhouse–Geisser correction: F(2.1, 81.9) = 89.4, *p* < 0.001, ε = 0.70, partial η^2^ = 0.70. Post hoc *p*-values from Bonferroni correction. CST, central subfield thickness; CI, confidence interval; ε, Greenhouse–Geisser epsilon.

## Data Availability

The original contributions presented in this study are included in the article. Further inquiries can be directed to the corresponding author.

## Abbreviations

The following abbreviations are used in this manuscript:

Abbreviation	Full Term
nAMD	Neovascular age-related macular degeneration
DME	Diabetic macular edema
CSR	Central serous retinopathy
CST	Central subfield thickness
IOP	Intraocular pressure
OCT	Optical coherence tomography
SD-OCT	Spectral-domain optical coherence tomography
anti-VEGF	Anti-vascular endothelial growth factor
ETDRS	Early Treatment Diabetic Retinopathy Study
ANOVA	Analysis of variance
CI	Confidence interval
SD	Standard deviation
